# Determining the efficacy of ginger *Zingiber officinale* as a potential nutraceutical agent for boosting growth performance and health status of *Labeo rohita* reared in a semi-intensive culture system

**DOI:** 10.3389/fphys.2022.960897

**Published:** 2022-08-15

**Authors:** Priya Rawat, Vaneet Inder Kaur, Anuj Tyagi, Parisa Norouzitallab, Kartik Baruah

**Affiliations:** ^1^ Department of Aquaculture, College of Fisheries, Guru Angad Dev Veterinary and Animal Sciences University (GADVASU), Ludhiana, PB, India; ^2^ Department of Aquatic Environment, College of Fisheries, GADVASU, Ludhiana, PB, India; ^3^ Department of Animal Nutrition and Management, Faculty of Veterinary Medicine and Animal Sciences, Swedish University of Agricultural Sciences, Uppsala, Sweden

**Keywords:** ginger, nutraceutical, *Labeo rohita*, sustainable carp culture, antibiotics

## Abstract

A 120-day feeding trial was conducted in a pilot field setting to study the nutraceutical properties of ginger powder (GP), focusing on the growth performance and health status of Indian major carp *L*. *rohita* reared under a semi-intensive culture system. *L. rohita* fingerlings (average weight: 20.5 g) were divided into five groups and fed a diet with no GP supplementation (control), or a diet supplemented with GP at 5 g (GP5), 10 g (GP10), 15 g (GP15), and 20 g (GP20) per kg of feed. The study was carried out in outdoor tanks (20 m^2^) following a complete randomized design with three replicates for each experimental group. Dietary supplementation of GP at 15 g·kg^−1^ (GP15) of feed caused a significant increase in the growth performances of the fish. Results also showed that feeding of GP15 diet led to a significant improvement in the health status of fish as indicated by a marked change in the tested haematological indices (i.e., higher RBC, WBC, Hb, and Ht values), oxidative status (increased SOD and decreased LPO levels), biochemical parameters (increased HDL, decreased cholesterol, and triglycerides levels), and activities of the liver enzymes (decreased AST and ALT). Overall results suggested that dietary supplementation of GP could positively influence the growth and health status of *L. rohita* fingerlings, and hence could be an important natural nutraceutical for sustainable farming of carp.

## Introduction

Freshwater aquaculture is an important source of protein worldwide. In South Asian countries, carps are the major freshwater cultured species, comprising around 85% of the total freshwater production for instance in India, contributing significantly to the economy and the reductions in poverty and nutritional insecurity. Among the three major Indian carps, *L. rohita* (commonly known as rohu) is the most preferred freshwater food species throughout the Indian subcontinent with a total production of 2.48 mmt (FAO, 2022). *L. rohita* is widely cultured under semi-intensive systems in India and its neighboring countries, with a yield of 3–5 tonnes·ha^−1^·year^−1^ (Rahman et al., 2006). In a semi-intensive pond-based aquaculture system, the dietary nutrient requirements of the farmed species are largely met through the consumption of natural food organisms produced endogenously within the pond and through the direct consumption of externally-supplied feed (Tacon and De Silva, 1997). Given the increasing demands on sustainable usage of primary resources, such as land and water, for the production of high-quality animal protein (Boyd et al., 2020), a great effort needs to be made to improve the semi-intensive farming systems, especially in the area of nutrition of fish farmed within such systems. Over the past few years, there has been a growing interest in using nutraceuticals, particularly those derived from sustainable and natural sources, in the fish feed to promote the growth performances and health status of farmed fishes, including carps (Mir et al., 2018) cultured in a semi-intensive pond farming system. The term nutraceutical is used to describe feed ingredients that demonstrate various health-beneficial effects in addition to their basic nutritional effects (Encarnação, 2016; Baruah et al., 2017). The multi-functional biological roles of the nutraceuticals for aquaculture species along with their easy accessibility, cost-effectiveness, and eco-friendly nature are some of the added advantages, which has encouraged their use in commercial aquaculture in different forms, i.e., whole plant, its parts (leaf, root or seed) or their extracts (Van Hai, 2015; Kaur et al., 2020).

Ginger (*Z*. *officinale*) is a creeping perennial underground rhizome belonging to the family *Zingiberaceae*, primarily grown in Asia (including India) and tropical regions, and is one of the most important and widely consumed herbs worldwide. Cultured for its edible underground stem (rhizome), ginger has been used since antiquity both as a condiment and as herbal medicine. The nonvolatile phenolic phytochemicals of ginger consist of gingerols, shogaols, paradols, and zingerone. Gingerols are a major active component in the fresh rhizome along with proteolytic enzyme namely Zingibain. These non-volatile components were reported to exhibit anti-inflammatory, immunological, and anti-bacterial bioactivities that are beneficial for the well-being of fish and shellfish (Ratna Shakya, 2015). In addition to its positive impact on health through its immune-enhancing effects, ginger has been proven to be a potent appetite enhancer and growth promoter due to its ability to improve the activity of digestive enzymes (Hassanin et al., 2014). A study carried out on juvenile common carp *Cyprinus carpio* had shown that ginger powder supplemented in the diet at a 2% inclusion level significantly enhanced the growth and biochemical composition of the fish (Abbasi Ghadikolaei et al., 2017). In another study on *Oreochromis niloticus*, feeding a diet supplemented with ginger at a 1% inclusion level was shown to cause a significant improvement in the growth performance and immune status of the fish (Hassanin et al., 2014). The beneficial effects of the ginger extract have also been demonstrated in rainbow trout *Oncorhynchus mykiss* (Dügenci et al., 2003) and Mozambique tilapia *Oreochromis mossambicus* (Immanuel et al., 2009), where feeding of a ginger extract supplemented diet at 1% inclusion level significantly improved many of the tested immune parameters i.e., lysozyme activity, total protein and globulin levels, phagocytic activity, and respiratory burst. Moreover, a challenge with *Vibrio vulnificus* showed an increase in the survival rate of Mozambique tilapia *O. mossambicus* fed a diet supplemented with ginger extract. In an earlier study on *L. rohita* fingerlings carried out under laboratory experimental conditions, Sukumaran et al. (2016) evaluated the nutraceuticals features of ginger powder focusing on the growth, skin mucus immune parameters, cytokine-related gene expression of *L. rohita*, and its resistance towards *Aeromonas hydrophila* bacterial challenge. After feeding for 60 days, the dietary ginger powder at a 0.8% inclusion level was shown to improve growth performance and skin mucosal immune response of *L. rohita*. Fish fed a diet with 0.8% ginger powder had the highest post-challenge survival, which was associated with the alteration in the expression of cytokine-related genes.

Until now, no validation study at least to our knowledge was conducted to determine the long-term effect of feeding ginger powder as a feed additive in the diet of *L. rohita* cultured under a semi-intensive system. Building upon the findings of Sukumaran et al. (2016), who examined the nutraceutical properties of ginger powder on *L. rohita* in a laboratory setting, we conducted a validation study by performing a nutritional experiment in a pilot field setting following semi-intensive farming practice to verify the nutraceutical properties of ginger.

## Materials and methods

### Experimental animal and maintenance

*L*. *rohita* fingerlings (*n* = 550) raised at the fish farm of the College of Fisheries, Guru Angad Dev Veterinary and Animal Sciences University, Punjab, India were used in this study. Before their use, the fingerlings were acclimatized to the experimental conditions for 15 days in 500-L tanks. During this period, they were fed a control diet at 3% of their body weight. After 15 days, the fingerlings with an average initial weight of 20.5 g were randomly distributed into five experimental groups with three replicates per group. Each group was maintained under an outdoor condition in a cemented experimental tank (20 m^2^) of 20,000-L water capacity at a density of 30 fish per tank. The bottom of the experimental tanks was covered with 1–2 inch soil to provide natural conditions. Lime was added at 300 kg·ha^−1^ for disinfection and balance of pH at the start of the experimental period. Water samples were collected fortnightly in the morning hours for the analyses of temperature, pH, dissolved oxygen, total alkalinity, total hardness, and ammonical nitrogen following standard protocols (APHA, 2012). The physicochemical parameters of rearing water viz. temperature (25.6–29.1°C), pH (7.2–8.6), dissolved oxygen (6.1–11.0 mg·L^−1^), total alkalinity (178.7–249.3 mg·L^−1^), total hardness (180.0–250.7 mg·L^−1^), and ammonia (0.009–0.09 mg·L^−1^) remained within the optimal range for carp culture throughout the experimental period (Boyd and Tucker, 1998).

### Preparation of ginger powder and experimental diets

Fresh rhizomes of ginger were obtained from the local market of the district Ludhiana, Punjab, India. They were washed thoroughly, peeled into thin pieces, and air-dried for 48 h in shade, ground into a fine powder using a mixer grinder, and sieved using a household sifter (2 mm). Ginger powder (GP) was mixed at different inclusion levels with other previously grounded feed ingredients, and five experimental diets were prepared. One with no GP (control) and the other four with GP at the inclusion level of 5 g (GP5), 10 g (GP10), 15 g (GP15), and 20 g (GP20) per kg of control diet (see Table 1, for details).

**TABLE 1:** Ingredients and formulation of the experimental diets for *L. rohita* used in this study.

Experimental diets				
Control	GP5	GP10	GP15	GP20
Basal diet^a^ with no GP supplementation	GP supplemented at 5 g·kg^−1^ of basal diet	GP supplemented at 10 g·kg^−1^ of basal diet	GP supplemented at 15 g·kg^−1^ of basal diet	GP supplemented at 20 g·kg^−1^ of basal diet

a Basal diet (g·kg^−1^): Rice bran (490 g) + Mustard meal (490 g) + Vitamin-mineral mixture (15 g)^b^ + Salt (5 g). GP, ginger powder.

b Each 250 g of vitamin-mineral mixture contains vitamin A - 500,000 IU, vitamin DS- 100,000 IU, vitamin B2-0.2 g, vitamin E - 75 units, vitamin K- 0.1 g, calcium pentathonate - 0.25 g, nicotinamide - 0.1 g, vitamin BI2 -0.6 mg, choline chloride -15 g, calcium -75 g, manganese -2.75 g, iodine -0.1 g, iron -0.75 g, zinc -1.5 g, copper -0.2 g, and cobalt -0.045 g.

The diets were prepared by mixing all the ingredients homogeneously followed by the addition of water to prepare the feed pellets through a hand pelletizer, which were dried at 60°C for 24 h and packed in air-tight containers for future use. The proximate compositions of the experimental diets (Table 2) were analyzed in triplicate following standard methods (AOAC, 2012). The fish in each group were fed twice daily (10:00 and 16:00 h) with ginger supplemented diets at 5% of the body weight for the first 60 days, and at 3% until day 120. The amount of feed given was adjusted after each sampling based on the increase in the fish weight.

**TABLE 2:** Proximate composition (on % dry matter basis) of different feed ingredients and experimental diets.

Ingredients/Diets	CP	EE	CF	NFE	Ash	Moisture	GE (K·cal·100 g^−1^)
Rice bran	15.4	2.1	23.6	46.9	11.9	3.1	299.5
Mustard meal	37.1	2.9	25.6	25.5	8.9	2.3	341.5
Ginger powder	5.6	2.5	5.0	81.7	5.2	4.6	390.2
Control	24.0	2.3	23.3	40.4	9.9	2.9	323.5
GP5	24.9	2.4	24.1	38.4	10.2	3.0	320.7
GP10	25.0	2.4	24.2	38.1	10.3	3.2	320.2
GP15	26.7	2.5	24.5	35.9	10.4	3.9	321.3
GP20	27.3	2.5	25.1	34.6	10.6	4.1	319.3

CP, Crude protein; EE, Ether extract; NFE, Nitrogen free extract; CF, Crude fat; GE, Gross energy.

### Experimental design

#### Growth performance

The survival and the growth performance of the fish were determined at the monthly interval following standard procedures (Halver, 1976):

Survival (%) = Fish harvested/Fish stocked × 100
Weight gain (%) = [Final body weight (g) − Initial body weight]/Initial body weight (g) × 100
Specific growth rate (SGR; % weight gain day^−1^) = logarithm final body weight (g) − logarithm initial body weight (g)/t (time interval in days) × 100
Apparent feed conversion ratio (AFCR) = Feed given (g)/Weight gain (g)
Apparent protein efficiency ratio (APER) = Weight gain (g)/Protein intake (g)

### Blood and serum collection

On day 60 and at the end (day 120) of the experiment, six fish were randomly sampled from each experimental tank and anesthetized with clove oil at 30–50 mg·L^−1^ (1 part clove oil and 9 parts 94% ethanol). Blood samples were collected from the caudal vein, using a 1.0 ml sterile disposable syringe, which was previously rinsed with heparin. For serum collection, the blood was collected without heparin and transferred to a 2 ml Eppendorf tube and kept for 6 h at room temperature followed by centrifugation at 2,200 g for 10 min. The supernatant was collected and stored at −20°C for the analysis of the haematological parameters, growth hormones, biochemical parameters, and liver enzymes.

### Preparation of haemolysate

Blood samples were centrifuged at 2,200 g for 15 min and the supernatant was separated. The sedimented cells were washed thrice with chilled 0.85% NaCl solution. Washed erythrocytes were lysed with nine parts of distilled water to prepare 10% haemolysate. Haemolysate was stored in aliquots at −20°C for the determination of oxidative stress markers.

### Analysis of hematological parameters and growth hormones

The red blood cells (RBC) and white blood cells (WBC) diluting fluids were used for determining RBC and WBC counts. Cell counts were performed using a haemocytometer. Hematocrit (Ht) was calculated following the method of (Mukherjee, 1988). The hemoglobin (Hb) concentration was estimated by the acid haematin method (Sahli, 1962). Mean corpuscular volume (MCV), mean corpuscular haemoglobin (MCH), and mean corpuscular haemoglobin concentration (MCHC) were calculated as previously described (Haney et al., 1992) using the following formulae:

$$\mathrm{MCV}\;\left({\mathrm{\mu m}}^{3}\right)\;=\;\mathrm{Ht}/\mathrm{RBC}\;\times \;10;\;\mathrm{MCH}\;\left(\mathrm{g\%}\right)\;=\;\mathrm{Hb}/\mathrm{RBC}\;\times \;10;\;\mathrm{MCHC}\;\left(\%\right)\;=\;\mathrm{MCH}/\mathrm{MCV}\;\times 100$$

Triiodothyronine (T3) and thyroxine (T4) were analyzed in the serum samples using commercial kits according to the manufacturer’s instructions (Erba Kits, Erba Mannheim, Germany)

### Biochemical analyses

The level of glucose, high-density lipid (HDL), total cholesterol, and triglycerides in the serum samples collected at the end of the feeding trial were analyzed according to the method of the International Federation of Clinical chemistry using commercial kits (Erba Mannheim, Germany).

### Analysis of liver enzymes

The activities of the alanine aminotransferase (ALT) and aspartate aminotransferase (AST) enzymes in the serum sample were analyzed following the method of the International Federation of Clinical chemistry using commercial kits (Erba Mannheim, Germany) (Fawole et al., 2017; Das et al., 2020).

### Stress indices

Lipid peroxidation (LPO) and superoxide dismutase (SOD) were analyzed on the blood haemolysate samples that were collected at the end of the feeding trial. The SOD activity was measured following the protocol of (Nishikimi et al., 1972). The assay is based on the principle that the nitroblue tetrazolium inhibits superoxide dismutase with reduced nicotinamide adenine dinucleotide (NADH) mediated by phenazonium methosulphate under aerobic conditions. One unit of SOD activity was defined as the amount of enzyme necessary to produce a 50% inhibition of the nitroblue tetrazolium reduction rate measured at 550 nm. SOD activity was expressed as U·mg·L^−1^·Hb. LPO assay is based on the reaction of malondialdehyde, an end product of lipid peroxidation with thiobarbituric acid to yield a pink-colored trimethine complex exhibiting an absorption maximum at 548 nm (Placer et al., 1966).

### Statistical analysis

Statistical analysis of the data was performed with a statistical package (SPSS 16.0 for Windows, SPSS Inc., Richmond, CA, United States). The data were analyzed using one-way ANOVA at a 5% significance level. The significant difference between the means was measured by Duncan’s multiple range test (Duncan, 1955).

## Results

### Survival and growth performance

No significant differences were observed in the survival of the fish fed different experimental diets, suggesting that ginger, at least within the inclusion levels tested, caused no adverse effect on the survival of *L. rohita* fingerlings. Dietary supplementation of GP at 15 g·kg^−1^ of diet (GP15) caused a significant improvement in the growth indices of *L. rohita* fingerlings as indicated by an increment in the value of WG percentage, SGR, and APER by 32%, 3.2%, and 27.4%, respectively as compared to control (Table 3). Compared to the control group as well as that fed GP5 or GP15 diet, the group fed the GP15 diet exhibited lower AFCR (20.9% lower as compared to the control group) value but the difference was not significant. However, the GP15 group had a significantly lower AFCR value than the GP20 group.

**TABLE 3:** Growth performance of the *L. rohita* after 120 days of feeding with diets supplemented with different levels of ginger powder.

Parameter	Control	GP5	GP10	GP15	GP20
Initial weight (g)	18.9 ± 3.4	21.3 ± 0.3	20.4 ± 0.2	20.6 ± 0.6	21.6 ± 0.8
Final weight (g)	130.1 ± 5.8^b^	136.5 ± 9.5^b^	132.4 ± 4.0^b^	157.0 ± 2.3^a^	110.4 ± 5.5^c^
Weight gain (%)	500.1 ± 29.8^bc^	542.6 ± 49.4^b^	550.4 ± 25.4^b^	662.3 ± 23.8^a^	412 ± 32.5^c^
SGR	2.5 ± 0.2^ab^	2.4 ± 0.1^ab^	2.4 ± 0.1^ab^	2.5 ± 0.1^a^	2.1 ± 0.1^b^
AFCR	2.0 ± 0.3^ab^	1.7 ± 0.2^ab^	1.9 ± 0.1^ab^	1.6 ± 0.2^b^	2.3 ± 0.2^a^
APER	1.6 ± 0.2^b^	1.6 ± 0.1^ab^	1.6 ± 0.2^ab^	2.0 ± 0.2^a^	1.4 ± 0.1^b^
Survival (%)	89.4 ± 1.5	92.4 ± 4.0	93.9 ± 1.5	96.9 ± 3.0	92.4 ± 1.5

SGR, Specific growth rate; AFCR, Apparent feed conversion ratio; APER, Apparent protein efficiency ratio.

These are significant differences among the treatments in a row.

### Haematological parameters

The haematological parameters of the fish like Hb, RBC, WBC, Ht, MCV, MCH, and MCHC were analyzed on day 60 and day 120 of the feeding period. A significant increase in the RBC count was observed in the GP15 group both on day 60 and day 120. The WBC count and the Hb level showed a significant increase in the GP5 and GP15 groups on day 60, and in the GP15 group on day 120 as compared to control and other treatment groups (Table 4). In comparison to the control, a significant improvement in the Ht value was observed in the GP5, GP10, and GP15 groups on day 60 and in the GP15 group on day 120. A significant variation in the MCV, MCH, and MCHC values was observed among the different experimental groups. A maximum MCV value was recorded in the GP5 group on day 60 as well as in the GP5 group on day 120, the value in the GP5 group, however, did not differ significantly from that in the GP10 group. On day 60, the MCH value was recorded highest in the GP20 group and was not significantly different from the control, GP5, and GP10 groups. On day 120, the MCH value was maximum in the GP5 group, however, the value did not vary significantly from the control, GP10, and GP20 groups. The MCHC values in the control and GP20 groups did not differ significantly on days 60 and 120. The values decreased significantly in the GP5, GP10, and GP15 groups in comparison to the control.

**TABLE 4:** Haematological parameters of *L. rohita* on day 60 and day 120 of the culture period in response to feeding diets supplemented with ginger powder at different inclusion levels.

Parameters	Days	Treatments*				
		Control	GP5	GP10	GP15	GP20
RBC (×10^6^ mm^3−1^)	60	1.4 ± 0.1^bc^	1.4 ± 0.1^bc^	1.6 ± 0.1^ab^	1.8 ± 0.1^a^	1.2 ± 0.1^c^
	120	1.7 ± 0.1^b^	1.5 ± 0.1^b^	1.6 ± 0.1^b^	2.3 ± 0.1^a^	1.7 ± 0.1^b^
WBC (×10^4^ mm^3−1^)	60	5.2 ± 0.2^b^	6.4 ± 0.2^a^	5.7 ± 0.2^b^	7.0 ± 0.3^a^	5.1 ± 0.1^b^
	120	5.7 ± 0.1^c^	6.8 ± 0.1^b^	5.9 ± 0.2^c^	8.5 ± 0.3^a^	5.6 ± 0.1^c^
Hb (g%)	60	7.9 ± 0.2^ab^	8.0 ± 0.1^a^	7.9 ± 0.2^ab^	8.3 ± 0.1^a^	7.5 ± 0.1^b^
	120	8.1 ± 0.3^b^	8.3 ± 0.1^b^	8.4 ± 0.3^b^	10.3 ± 0.5^a^	7.9 ± 0.1^b^
Ht/PCV (%)	60	19.2 ± 0.5^b^	33.8 ± 3.2^a^	27.4 ± 1.6^a^	28.1 ± 0.8^a^	20.0 ± 1.0^b^
	120	23.3 ± 1.0^c^	32.8 ± 1.1^b^	33.3 ± 1.9^b^	41.1 ± 0.5^a^	21.6 ± 1.0^c^
MCV (μ·m^3^)	60	133.1 ± 3.9^b^	246.8 ± 19.3^a^	172.7 ± 14.0^b^	154.4 ± 10.6^b^	167.9 ± 19.4^b^
	120	138.1 ± 5.9^c^	216.1 ± 1.3^a^	202.4 ± 6.3^ab^	178.4 ± 11.3^b^	128.7 ± 9.8^c^
MCH (g%)	60	54.6 ± 4.7^ab^	59.2 ± 3.9^a^	50.0 ± 3.0^ab^	45.8 ± 2.7^b^	62.3 ± 4.2^a^
	120	48.3 ± 3.7^ab^	54.8 ± 2.4^a^	51.4 ± 1.1^ab^	44.6 ± 2.8^b^	46.8 ± 1.6^ab^
MCHC (%)	60	40.9 ± 2.3^a^	24.3 ± 2.6^b^	29.1 ± 1.4^b^	29.7 ± 0.5^b^	37.6 ± 2.1^a^
	120	35.0 ± 2.6^a^	25.4 ± 1.1^b^	25.4 ± 0.4^b^	25.1 ± 1.6^b^	36.6 ± 1.5^a^

These are significant differences among the treatments in a row.

### Growth hormones

On day 60 of the feeding period, the dietary supplementation of GP caused no significant effect on the T3 hormone level. However, on day 120, the group fed the GP15 diet recorded the highest level of T3 compared to the control (Table 5). No significant difference in the level of T3 was recorded among the GP5, GP10, GP15, and GP20 groups. In the case of the T4 hormone, a maximum level of T4 was noted in the GP15 group, and the difference was significant when compared with the control (Table 5). On both days 60 and 120, the T4 level did not differ significantly between the groups fed GP-supplemented diets (Table 5).

**TABLE 5:** Thyroid hormones of *L. rohita* on day 60 and day 120 of the culture period in response to feeding diets supplemented with ginger powder at different inclusion levels.

Parameters	Days	Experimental groups				
		Control	GP5	GP10	GP15	GP20
Triiodothyronine (T3)	60	2.4 ± 0.1	2.4 ± 0.1	2.5 ± 0.1	2.5 ± 0.1	2.5 ± 0.1
	120	2.4 ± 0.1^b^	2.5 ± 0.1^ab^	2.5 ± 0.1^ab^	2.6 ± 0.1^a^	2.5 ± 0.2^a^
Thyroxine (T4)	60	1.2 ± 0.1^b^	1.2 ± 0.1^ab^	1.3 ± 0.1^ab^	1.4 ± 0.1^a^	1.3 ± 0.1^ab^
	120	1.2 ± 0.1^b^	1.3 ± 0.1^ab^	1.4 ± 0.1^a^	1.4 ± 0.1^a^	1.4 ± 0.1^a^

These are significant differences among the treatments in a row.

### Biochemical parameters

**FIGURE 1:**
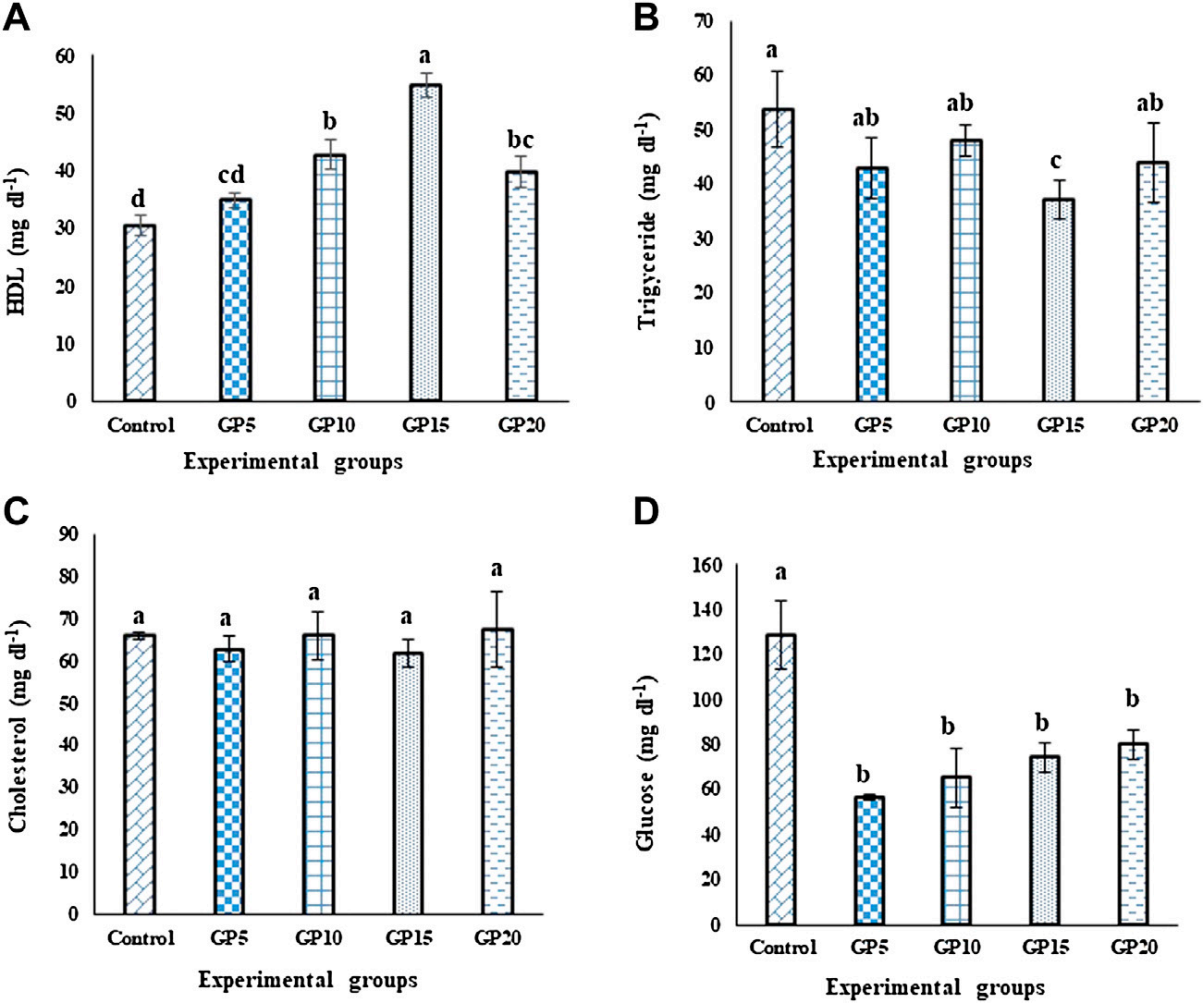
Effects of feeding various experimental diets for 120 days on the **(A)** high-density lipoprotein (HDL, mg·dl^−1^) **(B)** Triglycerides (mg·dl^−1^) **(C)** Cholesterol (mg·dl^−1^), and **(D)** Glucose (mg·dl^−1^) levels in the serum of *L. rohita*. Bars with different letters indicate significant differences (*p* < 0.05). Data are presented as mean ± standard error of three replicates.

Fish fed a GP15 diet for 120 days had the highest value of high-density lipids (HDL; Figure 1A), whereas the triglycerides value in this group was the lowest when compared with the control group (*p* ≤ 0.05; Figure 1B). The cholesterol level did not differ significantly among the different groups (Figure 1C). Dietary supplementation of GP caused a significant effect on the glucose level of the fingerlings, with the control group having the highest value as compared to all other ginger-fed groups (Figure 1D).

### Liver enzymes

**FIGURE 2:**
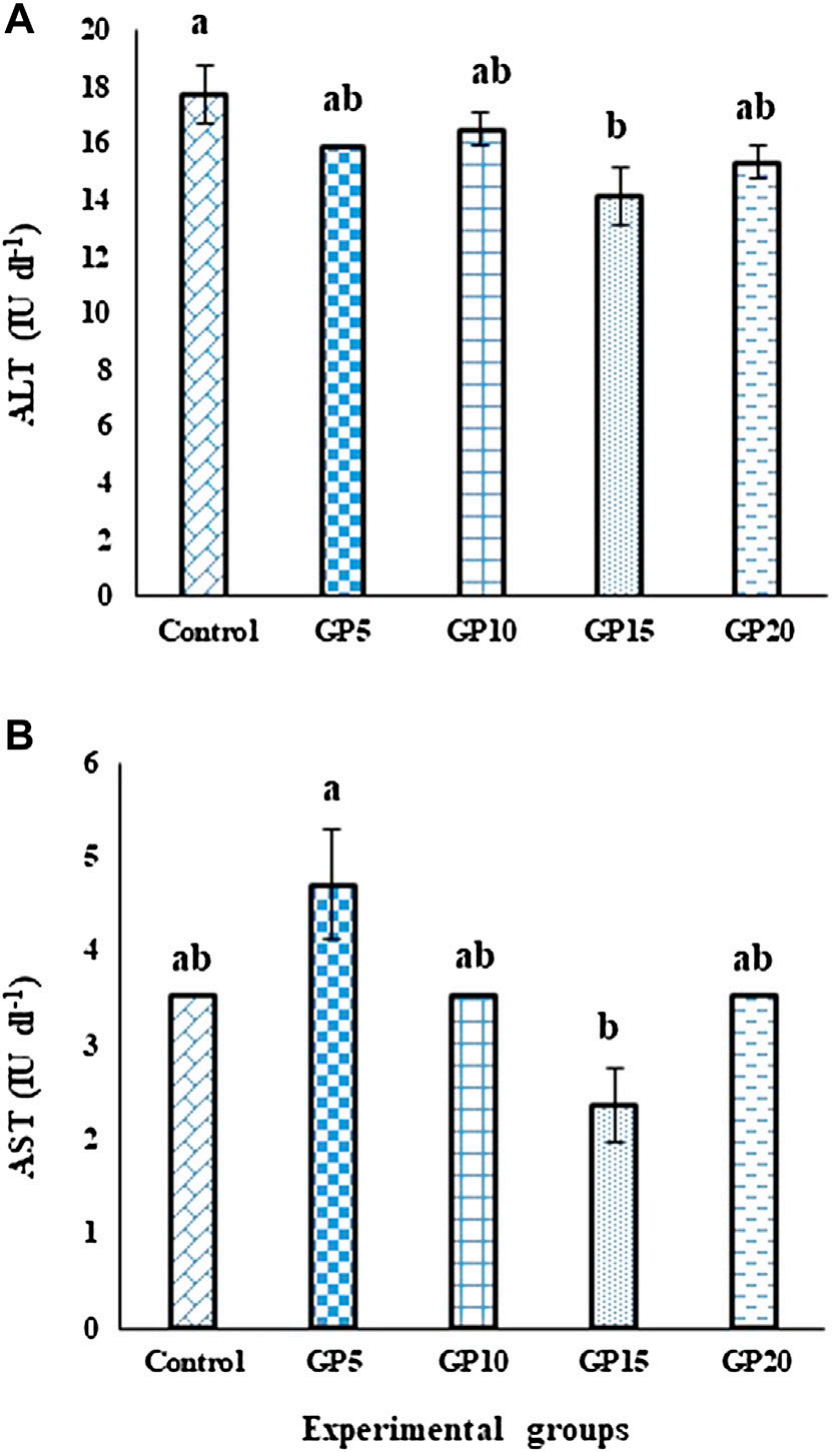
Effects of feeding various experimental diets for 120 days on the activities of **(A)** ALT (IU·dl^−1^) and **(B)** AST (IU·dl^−1^) enzymes in the serum of *L. rohita*. Bars with different letters indicate significant differences (*p* < 0.05). Data are presented as mean ± standard error of three replicates.

On day 120, the activity of the ALT enzyme in the GP15 group was significantly lower than control. However, the activity of the enzyme in this treatment group was not significantly different from the GP5, GP10, and GP20 groups (Figure 2A). In the case of the AST enzyme, the group fed the GP5 diet exhibited the highest enzyme activity, however, was not significantly different from the control group, as well as from the GP10 and GP20 groups. GP15 group showed the lowest enzyme activity and was not significantly different from the control, GP10, and GP20 groups (Figure 2B).

### Stress indices

**FIGURE 3:**
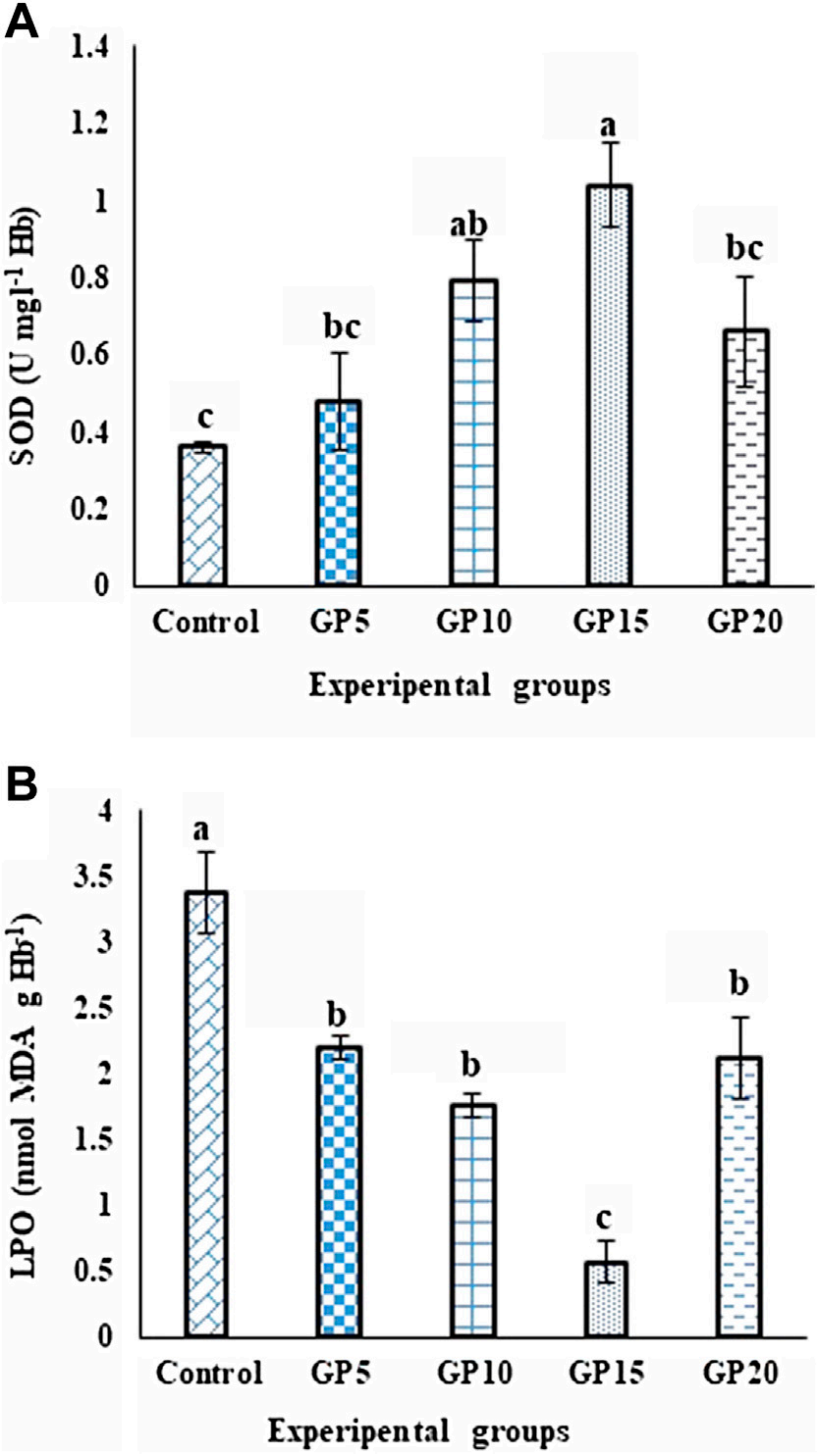
Effects of feeding various experimental diets for 120 days on the **(A)** SOD (U·mg·L^−1^·Hb) enzyme activity and **(B)** LPO (nmol·MDA·g·Hb^−1^) level in the serum of *L. rohita*. Bars with different letters indicate significant differences (*p* < 0.05). Data are presented as mean ± standard error of three replicates.

The activity level of the SOD enzyme in the fish fed GP-supplemented diets for 120 days increased with an increase in the inclusion level of GP in the diet, with the highest being recorded in the GP15 group. Increasing the inclusion level of GP to 20 g·kg^−1^ did not further increase the SOD activity level. In contrast, the enzyme activity level in the GP20 group significantly decreased when compared with the GP15 group (Figure 3A). The LPO value in the control group was the highest, whereas that in the GP15 group was recorded as the lowest, and this difference was significant when compared between the two groups, as with the GP5, GP10, and GP20 groups (Figure 3B).

## Discussion

Antibiotics have traditionally been used not only as therapeutic agents but also as growth promoters for improving the growth and health performances of farmed fish (Acar et al., 2000). Owing to the health hazards caused by the residues of the antibiotics on the fish as well as on the consumers, there has been an increasing effort in finding nutraceuticals from natural sources that aid in promoting fish growth and health in a sustainable manner. In this study, we investigated the long-term effect of feeding ginger powder (GP) as a source of nutraceuticals on the growth performance and health status of *L. rohita* fingerlings cultured under a semi-intensive aquaculture system. The GP was supplemented into a basal diet at different inclusion levels and each of these experimental diets was fed daily to the fish for 120 days. Our results showed that no significant mortality in response to feeding ginger-supplemented diet. From this result, it can be suggested that ginger caused no harmful effect on fish, at least in our described experimental condition. Hence, it can be considered safe for use in fish feed. Over the past few years, several researchers studied the beneficial effects of feeding ginger on aquaculture species but many of these studies were conducted in laboratory settings (Payung et al., 2017; Swain et al., 2018). In our study, we investigated the nutraceutical properties of GP in a pilot field setting by focusing on both the growth and health performances of the target animals. The basal diet was prepared following a standard feed formulation used by the carp farmers to contain an average of 25% CP and 2.5% lipid levels. The nutrient levels in the diets were maintained at relatively low assuming that the requirement will potentially be compensated by the naturally-grown planktons. The results showed that supplementation of ginger powder in the feed at 15 g·kg^−1^ caused a positive influence on the growth indices of *L. rohita* fingerlings. During the rearing period of 120 days, the weight gain in the group fed GP at 15 g·kg^−1^ of diet (i.e., GP15 group) increased by about 32% compared to the control group fed the basal diet. This resulted in an FCR that was significantly lower for the GP15 fed group in comparison to the other groups. The observed growth-promoting effects of the ginger-supplemented diet may be explained by the improved nutrient digestibility of experimental diets with ginger incorporation (Talpur et al., 2013; Hassanin et al., 2014). Moreover, the stimulated secretion of intestine protease enzyme because of feeding ginger, and its resulting impact on improving the digestion and absorption of protein components from the experimental feed could also be one of the underlying factors behind the observed increment in the growth performance of the fish. In line with the findings of our present study, previous studies also revealed the positive effect of dietary ginger (in powder or extract form) on the growth performances of many farmed fish species like *L. rohita* (Sukumaran et al., 2016), *C. carpio* (Jafarinejad et al., 2020), *O*. *mykiss* (Nya and Austin, 2009), and *L. calcarifer* (Talpur et al., 2013). It is also noteworthy to mention that ginger was shown to influence the growth of beneficial intestinal bacterial population, which aid in the utilization of nutrients (Hoseini et al., 2018) leading to the improvement of growth as observed in this study.

We cultured the fish under natural rearing conditions in outdoor cemented tanks covered with 1–2 inch soil to produce live feed. We did not measure the concentration of the planktons that were grown in the different experimental tanks. However, their contribution to the growth performances of the fish could not be excluded.

The health status of an organism plays a crucial role in its growth and development. It has been suggested that the well-being of fish is positively correlated with the effective utilization of nutrients in the diet and the conversion of these nutrients into tissue biomass (Sukumaran et al., 2016; Jafarinejad et al., 2020). In this study, we, therefore, examined whether feeding a ginger-supplemented diet could cause health benefits in the *L. rohita* fingerlings. To this end, we analyzed a range of blood parameters that can reflect fish health conditions and nutritional metabolism; factors useful for determining the health status of the fish in response to dietary supplements (Gabriel et al., 2015). Hematological parameters are an important physiological indicator in the prognosis of health-related disorders in fish and are influenced by a range of factors including species, size, age, diets, physiological status, and environmental conditions (Parma et al., 2007). In the present study, our results showed a significant improvement in the hematological parameters like RBC, WBC, Hb, and Ht in the fingerlings fed with a diet supplemented with 10 g or 15 g of GP as compared to the control. The increase in Hb level may be attributed due to the increase in the size of RBC (El-Feki et al., 1993), which in turn could enhance oxygen supply. The improved Hb content in GP15 demonstrates increased oxygen supply consequently, reflecting a beneficial effect on fish health. In agreement with our study, authors have reported higher values of Hb and WBCs in beluga *H. huso* due to feeding diet supplemented with ginger at an inclusion level of 10 g·kg^−1^ of feed (Vahedi et al., 2017). The results in terms of increased RBC count in GP15 are comparable to those, who reported that the RBC count was significantly higher in rainbow trout *O. mykiss* after feeding with ginger supplemented diet (10 g·kg^−1^ of diet) (Nya and Austin, 2009). The WBC serves as one of the first lines of body defense and their numbers increase sharply when infections arise. The increase in WBC counts along with other hematological parameters following the feeding of a ginger-supplemented diet suggests the immuno-stimulatory effects of ginger (Nya and Austin, 2009). The Ht index is the volumetric percentage of erythrocytes in systemic circulation and depends on the number and size of erythrocytes. Higher Ht reveals the ability of the blood to transport oxygen, which in turn indicates a healthy condition of fish (Birchard, 1997). The increased number and size of erythrocytes or Hb in the blood thus is accompanied by elevated Ht in the group fed a diet supplemented with 15 g·kg^−1^ of GP. The beneficial effects of ginger supplementation on RBC, Hb, and Ht have also been reported in previous studies on *L. calcarifer* (Talpur et al., 2013) and *O*. *mykiss* (Nya and Austin, 2009).

The thyroid hormones T4 and T3 play an important role in fish growth, development, metabolism, and reproduction (Power et al., 2001; Blanton and Specker, 2007). At the end of the feeding trial, the concentrations of T3 and T4 hormones in the serum were significantly elevated in ginger-fed fish as compared to the control. The positive correlation between the tested thyroid hormones and the growth performances in the ginger-fed group indicated the involvement of these hormones in the growth and development of fish. In line with our findings, earlier studies in fish highlighted the role of these hormones in regulating growth and development as well as metabolism (Quesada-García et al., 2014).

Ginger contains several biologically-active classes of phytochemicals (e.g., polyphenols, flavonoids, alkaloids, and saponins). Among them, several saponins have been reported to exhibit antihyperlipidemic activity (Talpur et al., 2013). In this study, one of the interesting observations that were made was the significant difference in the triglycerides level between the control and GP15 groups, with the maximum value of triglycerides in the control group and the lowest in the GP15 group. However, it is also noteworthy to mention that no significant difference in the triglyceride levels was found between other ginger-fed treatments and the control. Our results are in accordance with the finding of Vahedi et al. (2017), who reported a significant reduction in the value of triglycerides levels in beluga *Huso huso* after feeding 15 g·kg^−1^ of ginger extract as compared to the fish fed a control diet. The exact reason behind such an observation remains unclear. However, one can argue that the higher bioavailability of the saponins and/or possibly other classes of bioactive components (e.g., phlobatannin and anthraquinones) might have caused hypotriglyceridemic effects (Verma et al., 1993; Talpur et al., 2013), an assumption that warrants further studies.

In contrast to what was observed for triglycerides levels, the HDL content was recorded as highest in the GP15 groups and the lowest in the control group but the total cholesterol levels remained unaltered in response to feeding dietary ginger. A high HDL cholesterol level is an indicator of the good health status of the fish, as it is known as good cholesterol because it helps to remove other forms of unhealthy cholesterol from the bloodstream (Gabriel et al., 2015). In line with our findings, higher serum HDL was observed in red sea bream *Pagrus major* fed a diet supplemented with *Cnoglossum officinale* (Ji et al., 2007) and in *O. niloticus* fed with *Aloe vera* (Gabriel et al., 2015).

The present study further demonstrated that the ginger-supplemented diet significantly decreased the blood glucose level in *L. rohita* compared to the control fish. The observed response is an indication that ginger, possibly through its bioactive compounds (such as gingerols, shogaols, paradols, and zingerone), could have enhanced the level of insulin during the course of the 120-day administration, leading to a decrease in blood glucose level (Sahu et al., 2007; Farahi et al., 2010). The findings of the present study in terms of decreased glucose level are in close agreement with the findings of previous studies conducted on *L. rohita* and Asian sea bass *Lates calcarifer* fed with garlic-supplemented diets (Sahu et al., 2007; Talpur et al., 2013).

Liver enzymes ALT and AST are one of the most commonly measured biomarkers for fish health (Canli et al., 2018). Significantly elevated levels of these enzymes indicate the existence of health problems, such as degeneration, necrosis, and destruction of the liver due to cellular damage (Bhardwaj et al., 2010). In this study, we did not observe a significant increase in the activities of ALT and AST enzymes in any of the ginger-fed groups. In fact, the GP15 group that showed the best growth performances exhibited lower activities of ALT and AST when compared with the control group. The observed effect in the GP15 group could be explained by the bioavailability of different classes of bioactive components from the ginger, possibly in the optimal level, which might have aided in the smooth functioning of the liver and in the protection against any possible cellular damage. The results of this study are in accordance with the findings that reported a lowering of liver enzymes in juvenile beluga, when fed with ginger-supplemented diets (Vahedi et al., 2017).

The improvement of antioxidant and hepatoprotective parameters in fish by functional plant ingredients is mediated by the nutraceuticals available in the ingredients (Goda, 2008). The majority of the active ingredients like gingerols, shogaols, and zingerone present in ginger are known to possess antioxidant activity, with a high capacity to scavenge free radicals (Chrubasik et al., 2005). These bioactive components in ginger are believed to enhance the body’s natural antioxidant against oxidative stress by increasing the amount and activity of the body’s natural antioxidant enzymes, such as liver SOD (Rajasekaran et al., 2005). According to the obtained results, the activity of the SOD enzyme was significantly elevated in the GP15 group in comparison to the control group. Such an elevation might have resulted in causing lipid peroxidation inhibition in the ginger-fed groups, with the most prominent being observed in the GP15 group. Authors have reported high activity of SOD in sobaity sea bream *Sparidentex hasta* when fed with diets supplemented with 0.1 g ginger kg^−1^ of feed (Jahanjoo et al., 2018). In another study on *C. carpio*, feeding of a diet supplemented with ginger extract at a dose of 2 g·kg^−1^ caused a significant improvement in the SOD activity (Mohammadi et al., 2020). Furthermore, (Şahan et al., 2016; Mahmoud et al., 2019), in Nile tilapia reported that ginger powder exhibited antioxidant activity against free radicals and significantly decreased the LPO level in the fish.

## Conclusion

In essence, our results showed that dietary GP at 15 g·kg^−1^ of feed improved the growth performance and health status of *L. rohita* fingerlings cultured under a semi-intensive farming system. Due to the easy availability of this natural additive, it can be recommended to be used as a nutraceutical agent in the diet of rohu for its sustainable production. The modes of action of dietary GP in promoting growth performances and health status remained unclear. It is very likely several bioactive components available in the ginger possibly interact in a synergistic and/or additive manner to cause these beneficial effects. Further research needs to be carried out to study the underlying mechanism of action of ginger in *L. rohita*. Further studies are also needed for the effective use of this functional ingredient in other commercially important aquaculture species.

## Data Availability

The raw data supporting the conclusion of this article will be made available by the authors, without undue reservation.

## References

[B1] Abbasi Ghadikolaei H. Kamali A. Soltani M. Sharifian M. (2017). Effects of Zingiber officinale powder on growth parameters, survival rate and biochemical composition of body in juvenile common carp (*Cyprinus carpio*). Iran. J. Fish. Sci. 16, 67–85.

[B2] Acar J. Casewell M. Freeman J. Friis C. Goossens H. (2000). Avoparcin and virginiamycin as animal growth promoters: A plea for science in decision-making. Clin. Microbiol. Infect. 6, 477–482. 10.1046/j.1469-0691.2000.00128.x 11168181

[B3] APHA (2012). Standards methods for the examination of water and wastewater. 22nd ed. Washington, DCUSA.

[B4] Association of Official Analytical Chemists (2012). Official Methods of Analysis of AOAC INTERNATIONAL. Washington D.C. USA: Aoac 19st.

[B5] Baruah K. Norouzitallab P. Phuong H. Duy P. (2017). Enhanced resistance against Vibrio harveyi infection by carvacrol and its association with the induction of heat shock protein 72 in gnotobiotic Artemia franciscana. 10.1007/s12192-017-0775-z PMC5425368 28303510

[B6] Bhardwaj S. Srivastava M. K. Kapoor U. Srivastava L. P. (2010). A 90 days oral toxicity of imidacloprid in female rats: Morphological, biochemical and histopathological evaluations. Food Chem. Toxicol. 48, 1185–1190. 10.1016/J.FCT.2010.02.009 20146932

[B7] Birchard G. F. (1997). Optimal hematocrit: Theory, regulation and implications. Am. Zool. 37, 65–72. 10.1093/ICB/37.1.65

[B8] Blanton M. L. Specker J. L. (2007). The hypothalamic-pituitary-thyroid ( HPT ) Axis in fish and its role in fish development and reproduction. 97–115. 10.1080/10408440601123529 17364706

[B9] Boyd C. E. D’Abramo L. R. Glencross B. D. Huyben D. C. Juarez L. M. Lockwood G. S. (2020). Achieving sustainable aquaculture: Historical and current perspectives and future needs and challenges. J. World Aquac. Soc. 51, 578–633. 10.1111/JWAS.12714

[B10] Boyd C. E. Tucker C. S. (1998). Pond aquaculture water quality management. 10.1007/978-1-4615-5407-3

[B11] Canli E. G. Dogan A. Canli M. (2018). Serum biomarker levels alter following nanoparticle (Al2O3, CuO, TiO2) exposures in freshwater fish (*Oreochromis niloticus*). Environ. Toxicol. Pharmacol. 62, 181–187. 10.1016/J.ETAP.2018.07.009 30053707

[B12] Chrubasik S. Pittler M. H. Roufogalis B. D. (2005). Zingiberis rhizoma: A comprehensive review on the ginger effect and efficacy profiles. Phytomedicine 12, 684–701. 10.1016/J.PHYMED.2004.07.009 16194058

[B13] Das P. Das M. Kalita A. Chutia P. (2020). Studies on toxicological effect of the herbicide paraquat dichloride on the air breathing singhi catfish, heteropneustes fossilis (bloch). Proc. Zool. Soc. 73, 406–417. 10.1007/s12595-020-00346-2

[B14] Dügenci S. K. Arda N. Candan A. (2003). Some medicinal plants as immunostimulant for fish. J. Ethnopharmacol. 88, 99–106. 10.1016/S0378-8741(03)00182-X 12902058

[B15] Duncan D. B. (1955). Multiple range and multiple F tests. Biometrics 11, 1. 10.2307/3001478

[B16] El-Feki M. A. Tawfek N. S. Awad E. M. (1993). Ulcerative dermal necrosis, a freshwater fish disease and haematological studies. Journal of the Egyptian Society of Toxicology. J. Egypt. Soc. Toxicol. 10, 25–28.

[B17] Encarnação P. (2016). Functional feed additives in aquaculture feeds. Aquafeed Formul., 217–237. 10.1016/B978-0-12-800873-7.00005-1

[B18] FAO (2022). The state of world Fisheries and aquaculture 2022. Towards blue transformation. Rome: FAO. 10.4060/cc0461en

[B19] Farahi A. Kasiri M. Sudagar M. Iraei M. S. Shahkolaei M. D. (2010). Effect of garlic ( Allium sativum ) on growth factors, some hematological parameters and body compositions in rainbow trout ( *Oncorhynchus mykiss* ). Aquaculture, Aquarium, Conservation and Legislation Bioflux , 3. Aquac. Aquar. Conserv. Legis. Bioflux 3, 317317–323323.

[B20] Fawole F. J. Sahu N. P. Jain K. K. Gupta S. Rajendran K. V. Shamna N. (2017). Haemato-biochemical, non-specific immunity, antioxidant capacity and histopathological changes in *Labeo rohita* fingerlings fed rubber protein isolate. Fish. Physiol. Biochem. 43, 677–690. 10.1007/s10695-016-0322-3 27957678

[B21] Gabriel N. N. Qiang J. Ma X. Y. He J. Xu P. Liu K. (2015). Dietary Aloe vera improves plasma lipid profile, antioxidant, and hepatoprotective enzyme activities in GIFT-tilapia (*Oreochromis niloticus*) after Streptococcus iniae challenge. Fish. Physiol. Biochem. 41, 1321–1332. 10.1007/s10695-015-0088-z 26109009

[B22] Goda A. M. A. S. (2008). Effect of dietary ginseng herb (Ginsana® G115) supplementation on growth, feed utilization, and hematological indices of nile Tilapia, *Oreochromis niloticus* (L.), fingerlings. J. World Aquac. Soc. 39, 205–214. 10.1111/J.1749-7345.2008.00153.X

[B23] Halver J. E. (1976). “The nutritional requirements of cultivated warm water and cold water fish species,” in FAO Technical Conference on Aquaculture, Kyoto, Japan.9

[B24] Haney D. C. Hursh D. A. Mix M. C. Winton J. R. (1992). Physiological and hematological changes in chum salmon artificially infected with erythrocytic necrosis virus. J. Aquat. Anim. Health 4, 48–57. 10.1577/1548-8667(1992)004<0048:pahcic>2.3.co;2

[B25] Hassanin M. El-S. Hakim Y. Badawi M. E.-S. (2014). Dietry effect of ginger ( zingiber officinale roscoe ) on growth performance , immune response of nile Tilapia ( Oreochromis niloticus ) and disease resistance against aeromonas hydroph. Abassa Int. J. Aquac. 7, 35–52.

[B26] Hoseini S. M. Taheri Mirghaed A. Iri Y. Ghelichpour M. (2018). Effects of dietary cineole administration on growth performance, hematological and biochemical parameters of rainbow trout (*Oncorhynchus mykiss*). Aquaculture 495, 766–772. 10.1016/J.AQUACULTURE.2018.06.073

[B27] Immanuel G. Uma R. P. Iyapparaj P. Citarasu T. Punitha Peter S. M. Michael Babu M. (2009). Dietary medicinal plant extracts improve growth, immune activity and survival of tilapia *Oreochromis mossambicus* . J. Fish. Biol. 74, 1462–1475. 10.1111/j.1095-8649.2009.02212.x 20735646

[B28] Jafarinejad R. Gharaei A. Mirdar Harijani J. (2020). Dietary ginger improve growth performance, blood parameters, antioxidant capacity and gene expression in *Cyprinus carpio* . Iran. J. Fish. Sci. 19, 1237–1252. 10.22092/ijfs.2018.119876

[B29] Jahanjoo V. Yahyavi M. Akrami R. Bahri A. H. (2018). Influence of Adding Garlic (Allium sativum), Ginger (Zingiber officinale), Thyme (Thymus vulgaris) and Their Combination on the Growth Performance, HaematoImmunological Parameters and Disease Resistance to Photobacterium damselae in Sobaity Sea Bream (Sparidentex hasta) Fry. Turk. J. Fish. Aquat. Sci. 18, 633–645. 10.4194/1303-2712-V18_4_15

[B30] Ji S. Takaoka O. Jeong G. S. Lee S. W. Ishimaru K. Seoka M. (2007). Dietary medicinal herbs improve growth and some non-specific immunity of red sea bream *Pagrus major* . Fish. Sci. 73, 63–69. 10.1111/j.1444-2906.2007.01302.x

[B31] Kaur A. Shanthanagouda A. H. Kaur V. I. Bansal N. Billekallu Thammegowda N. K. (2020). Biochemical and histomorphological associated *in vivo* responses of turmeric supplemented diets in Rohu, Labeo rohita (Linn.). Aquac. Res. 51, 3915–3923. 10.1111/ARE.14740

[B32] Mahmoud R. Aziza A. Marghani B. Eltaysh R. (2019). Influence of ginger and garlic supplementation on growth performance, whole body composition and oxidative stress in the muscles of nile tilapia (*O. niloticus*). Adv. Anim. Vet. Sci. 7, 397–404. 10.17582/journal.aavs/2019/7.5.397.404

[B33] Mir I. N. Sushila N. Ahmad B. I. Ahmad D. S. Dar Jaffer Yousf A. P. M. Syamala K. (2018). Fucoidan: A sulphated polysaccharide and its bioactive potential in aquaculture. Aquac. Times, 17–21. Available at: https://www.researchgate.net/publication/327052618 (Accessed July 1, 2022).

[B34] Mohammadi G. Rashidian G. Hoseinifar S. H. Naserabad S. S. Doan H. V. (2020). Ginger (Zingiber officinale) extract affects growth performance, body composition, haematology, serum and mucosal immune parameters in common carp (*Cyprinus carpio*). Fish. Shellfish Immunol. 99, 267–273. 10.1016/j.fsi.2020.01.032 31981777

[B35] Mukherjee K. L. (1988). Medical laboratory Technology, 1. New Delhi: Tata Mc-Graw hill publishing company Ltd.

[B36] Nishikimi M. Appaji Rao N. Yagi K. (1972). The occurrence of superoxide anion in the reaction of reduced phenazine methosulfate and molecular oxygen. Biochem. Biophys. Res. Commun. 46, 849–854. 10.1016/S0006-291X(72)80218-3 4400444

[B37] Nya E. J. Austin B. (2009). Use of dietary ginger, Zingiber officinale Roscoe, as an immunostimulant to control Aeromonas hydrophila infections in rainbow trout, *Oncorhynchus mykiss* (Walbaum). J. Fish. Dis. 32, 971–977. 10.1111/j.1365-2761.2009.01101.x 19843197

[B38] Parma M. J. Loteste A. Campana M. Bacchetta C. (2007). Changes of hematological parameters in *Prochilodus lineatus* (Pisces, Prochilodontidae) exposed to sublethal concentration of cypermethrin. J. Environ. Biol. 28, 147–149. 17718004

[B39] Payung C. N. Tumbol R. A. Manoppo H. (2017). Dietary ginger (Zingiber officinale) enhance resistance of Nile tilapia (*Oreochromis niloticus*) against Aeromonas hydrophila. AACL Bioflux 10.

[B40] Placer Z. A. Cushman L. L. Johnson B. C. (1966). Estimation of product of lipid peroxidation (malonyl dialdehyde) in biochemical systems. Anal. Biochem. 16, 359–364. 10.1016/0003-2697(66)90167-9 6007581

[B41] Power D. M. Llewellyn L. Faustino M. Nowell M. A. Björnsson B. T. Einarsdottir I. E. (2001). Thyroid hormones in growth and development of fish. Comp. Biochem. Physiol. C. Toxicol. Pharmacol. 130, 447–459. 10.1016/S1532-0456(01)00271-X 11738632

[B42] Quesada-García A. Valdehita A. Kropf C. Casanova-Nakayama A. Segner H. Navas J. M. (2014). Thyroid signaling in immune organs and cells of the teleost fish rainbow trout (*Oncorhynchus mykiss*). Fish. Shellfish Immunol. 38, 166–174. 10.1016/j.fsi.2014.03.016 24657316

[B43] Rahman M. H. Arifuzzaman M. (2021). An experiment on growth performance, specifi c growth rate (SGR) and feed conversion ratio (FCR) of rohu (Labeo rohita) and Tilapia (*Oreochromis niloticus*) in tank based intensive aquaculture system. Int. J. Aquac. Fish. Sci. 07, 35–41. 10.46505/ijbi.2021.3208

[B44] Rahman M. M. Verdegem M. C. J. Nagelkerke L. A. J. Wahab M. A. Milstein A. Verreth J. A. J. (2006). Growth, production and food preference of rohu *Labeo rohita* (H.) in monoculture and in polyculture with common carp *Cyprinus carpio* (L.) under fed and non-fed ponds. Aquaculture 257, 359–372. 10.1016/j.aquaculture.2006.03.020

[B45] Rajasekaran S. Sivagnanam K. Subramanian S. (2005). Antioxidant effect of Aloe vera gel extract in streptozotocin-induced diabetes in rats. Pharmacol. Rep. 57, 90–96. 15849382

[B46] Ratna Shakya S. (2015). Medicinal uses of ginger (Zingiber officinale Roscoe) improves growth and enhances immunity in aquaculture. Int. J. Chem. Stud. 3.

[B47] Şahan A. Özütok S. Kurutaş E. B. (2016). Determination of some hematological parameters and antioxidant capacity in Nile tilapia (Oreochromis Niloticus Linnaeus, 1758) fed ginger (zingiber officinale roscoe) to aeromonas hydrophila. Turk. J. Fish. Aquat. Sci. 16, 197–204. 10.4194/1303-2712-v16_1_20

[B48] Sahli T. (1962). in Textbook of chemical pathology. Editor Scward E. (Baltimore, USA: Williams and Williams C.).

[B49] Sahu B. S. Das B. K. Mishra B. K. Pradhan J. Sarangi N. (2007). Effect of Allium sativum on the immunity and survival of *Labeo rohita* infected with *Aeromonas hydrophila* . J. Appl. Ichthyol. 23, 80–86. 10.1111/j.1439-0426.2006.00785.x

[B50] Sukumaran V. Park S. C. Giri S. S. (2016). Role of dietary ginger Zingiber officinale in improving growth performances and immune functions of Labeo rohita fingerlings. Fish. Shellfish Immunol. 57, 362–370. 10.1016/J.FSI.2016.08.056 27574828

[B51] Swain S. Pawase A. S. Pai R. K. Tibile R. M. Indulkar S. T. Pawar R. A. (2018). Effect of ginger ( Zingiber officinale Roscoe ) incorporated diet on growth performance of striped catfish , Pangasianodon hypophthalmus. J. Entomol. Zool. Stud. 6, 1094–1098.

[B52] Tacon A. G. J. De Silva S. S. (1997). Feed preparation and feed management strategies within semi-intensive fish farming systems in the tropics. Aquaculture 151, 379–404. 10.1016/S0044-8486(96)01494-9

[B53] Talpur A. D. Ikhwanuddin M. Ambok Bolong A. M. (2013). Nutritional effects of ginger (*Zingiber officinale Roscoe*) on immune response of Asian sea bass, *Lates calcarifer* (Bloch) and disease resistance against Vibrio harveyi. Aquaculture 401, 46–52. 10.1016/j.aquaculture.2013.02.043

[B54] Vahedi A. Hasanpour M. Akrami R. Chitsaz H. Department of Fisheries, Azadshahr Branch, Islamic Azad University, Azadshahr, Iran, Department of Fisheries, Khazar Institue of Higher Education, Mahmoud Abad, Iran (2017). Effect of dietary supplementation with ginger (Zingiber officinale) extract on growth, biochemical and hemato-immunological parameters in juvenile beluga (*Huso huso*). I. J. Aqua. Anim. Health 3, 26–46. 10.18869/acadpub.ijaah.3.1.26

[B55] Van Hai N. (2015). The use of medicinal plants as immunostimulants in aquaculture: A review. Aquaculture 446, 88–96. 10.1016/J.AQUACULTURE.2015.03.014

[B56] Verma S. K. Singh J. Khamesra R. Bordia A. (1993). Effect of ginger on platelet aggregation in man. Indian J. Med. Res. 98, 240–242. Available at: https://europepmc.org/article/med/8119760 (Accessed May 20, 2022). 8119760

